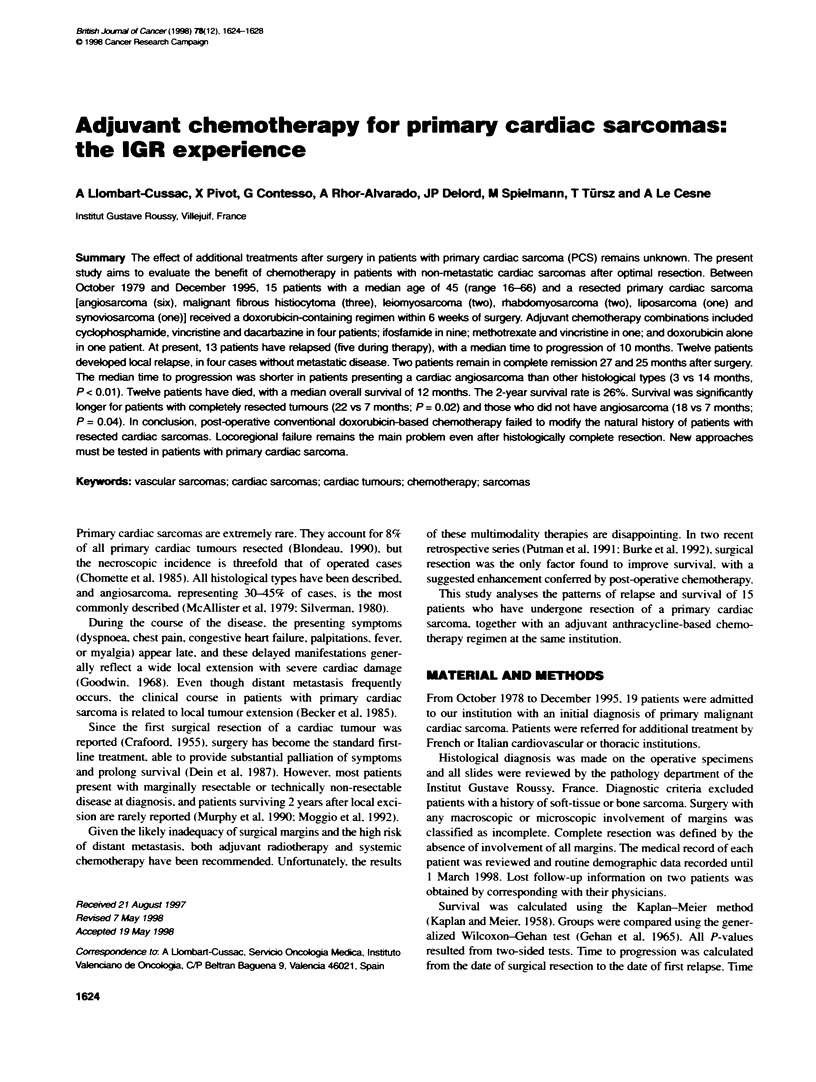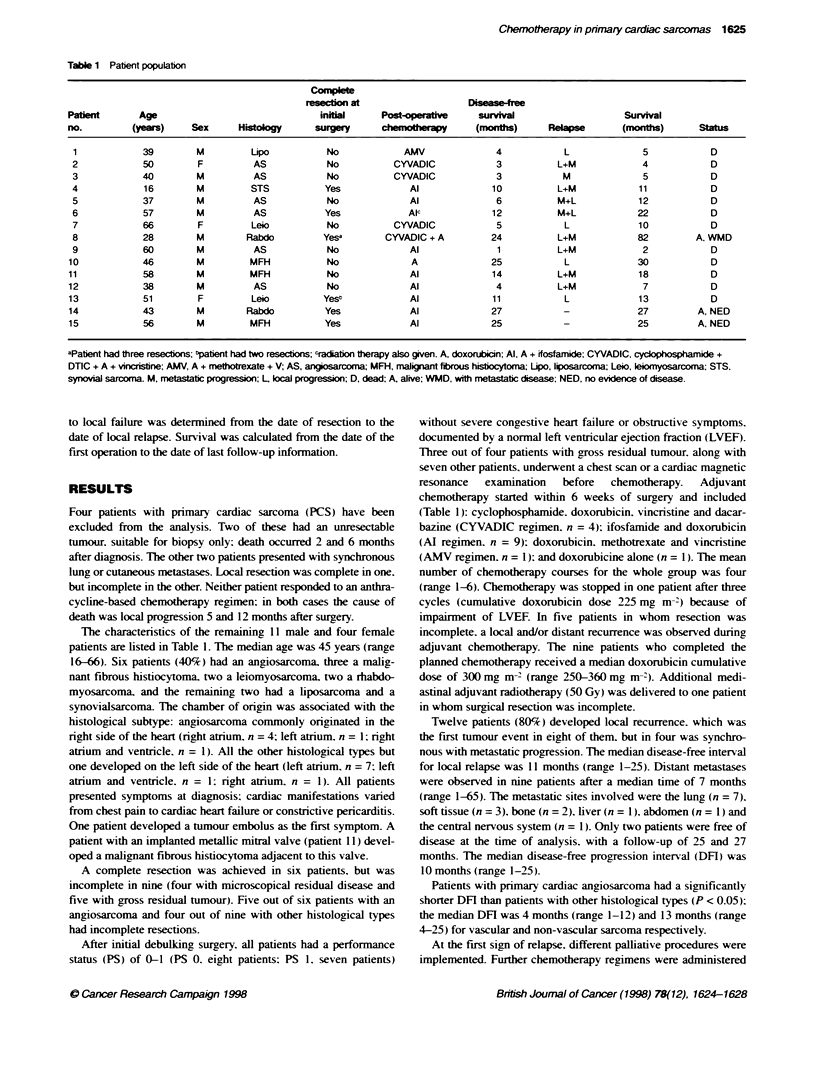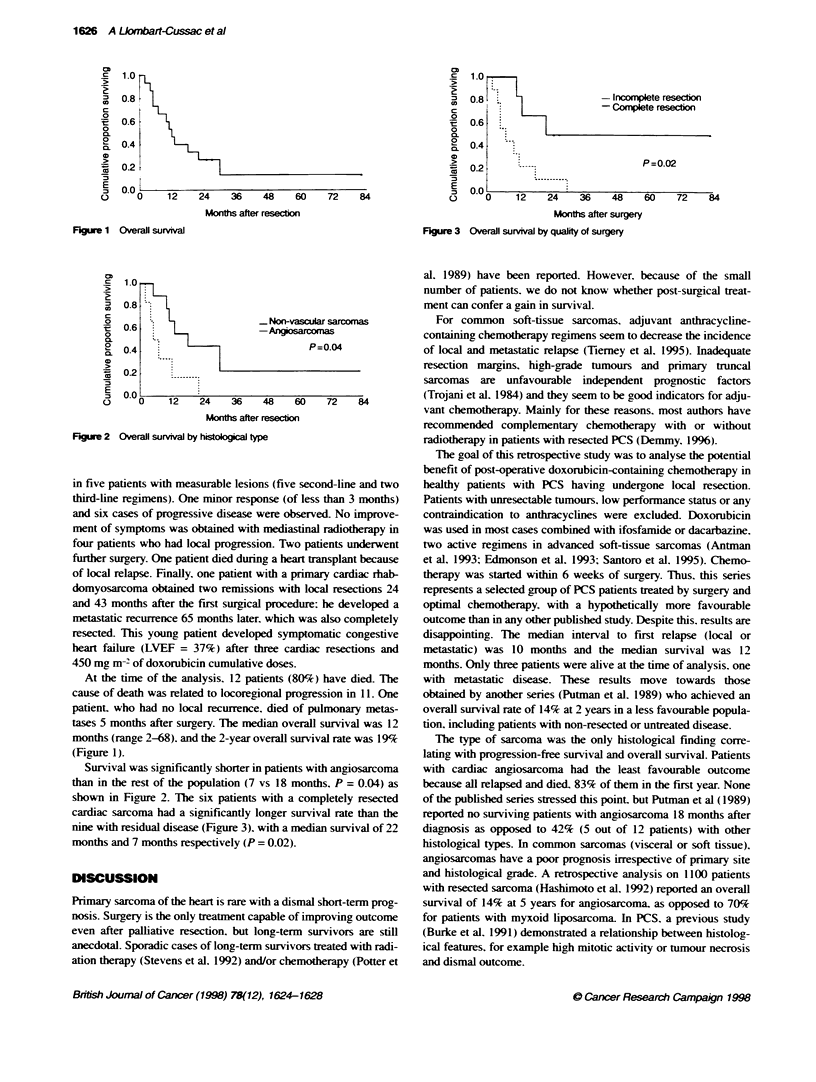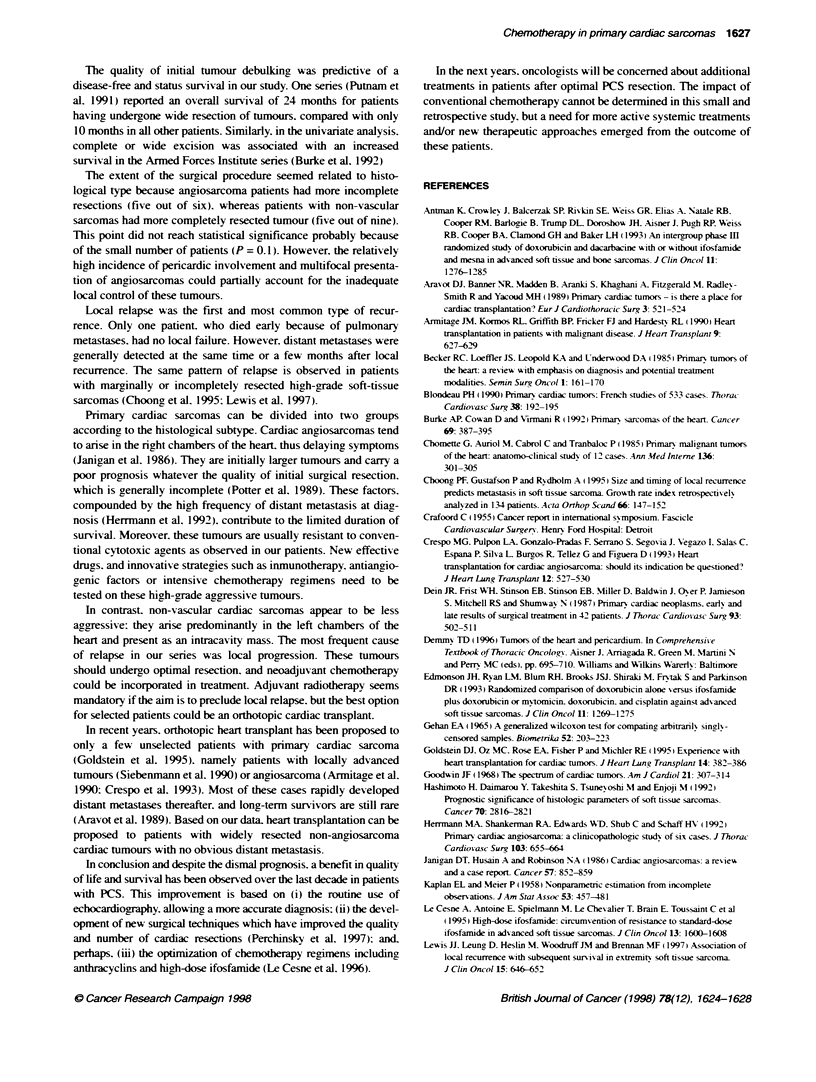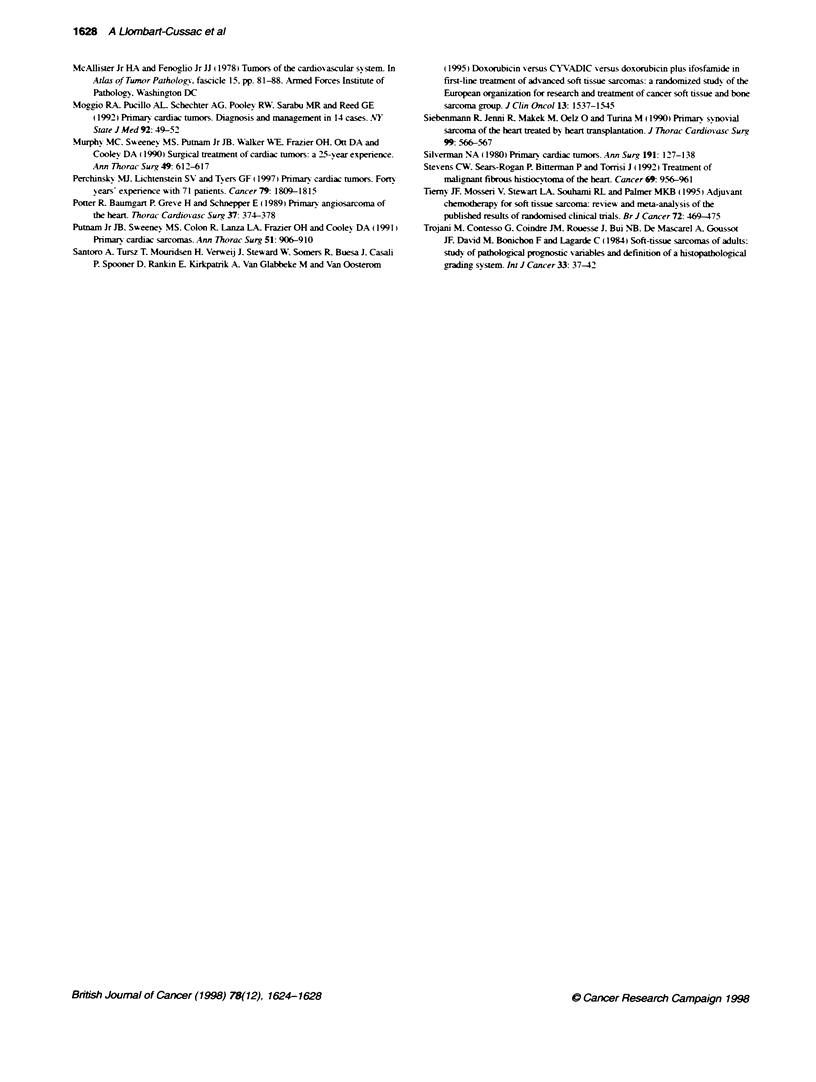# Adjuvant chemotherapy for primary cardiac sarcomas: the IGR experience.

**DOI:** 10.1038/bjc.1998.733

**Published:** 1998-12

**Authors:** A. Llombart-Cussac, X. Pivot, G. Contesso, A. Rhor-Alvarado, J. P. Delord, M. Spielmann, T. Türsz, A. Le Cesne

**Affiliations:** Institut Gustave Roussy, Villejuif, France.

## Abstract

The effect of additional treatments after surgery in patients with primary cardiac sarcoma (PCS) remains unknown. The present study aims to evaluate the benefit of chemotherapy in patients with non-metastatic cardiac sarcomas after optimal resection. Between October 1979 and December 1995, 15 patients with a median age of 45 (range 16-66) and a resected primary cardiac sarcoma [angiosarcoma (six), malignant fibrous histiocytoma (three), leiomyosarcoma (two), rhabdomyosarcoma (two), liposarcoma (one) and synoviosarcoma (one)] received a doxorubicin-containing regimen within 6 weeks of surgery. Adjuvant chemotherapy combinations included cyclophosphamide, vincristine and dacarbazine in four patients; ifosfamide in nine; methotrexate and vincristine in one; and doxorubicin alone in one patient. At present, 13 patients have relapsed (five during therapy), with a median time to progression of 10 months. Twelve patients developed local relapse, in four cases without metastatic disease. Two patients remain in complete remission 27 and 25 months after surgery. The median time to progression was shorter in patients presenting a cardiac angiosarcoma than other histological types (3 vs 14 months, P < 0.01). Twelve patients have died, with a median overall survival of 12 months. The 2-year survival rate is 26%. Survival was significantly longer for patients with completely resected tumours (22 vs 7 months; P = 0.02) and those who did not have angiosarcoma (18 vs 7 months; P = 0.04). In conclusion, post-operative conventional doxorubicin-based chemotherapy failed to modify the natural history of patients with resected cardiac sarcomas. Locoregional failure remains the main problem even after histologically complete resection. New approaches must be tested in patients with primary cardiac sarcoma.


British Journal of Cancer (1998) 78(12), 1624–1628
© 1998 Cancer Research Campaign

### Adjuvant chemotherapy for primary cardiac sarcomas: the IGR experience

**A Llombart-Cussac, X Pivot, G Contesso, A Rhor-Alvarado, JP Delord, M Spielmann, T Türsz and A Le Cesne**

Institut Gustave Roussy, Villejuif, France

**Summary** The effect of additional treatments after surgery in patients with primary cardiac sarcoma (PCS) remains unknown. The present study aims to evaluate the benefit of chemotherapy in patients with non-metastatic cardiac sarcomas after optimal resection. Between October 1979 and December 1995, 15 patients with a median age of 45 (range 16–66) and a resected primary cardiac sarcoma [angiosarcoma (six), malignant fibrous histiocytoma (three), leiomyosarcoma (two), rhabdomyosarcoma (two), liposarcoma (one) and synoviosarcoma (one)] received a doxorubicin-containing regimen within 6 weeks of surgery. Adjuvant chemotherapy combinations included cyclophosphamide, vincristine and dacarbazine in four patients; ifosfamide in nine; methotrexate and vincristine in one; and doxorubicin alone in one patient. At present, 13 patients have relapsed (five during therapy), with a median time to progression of 10 months. Twelve patients developed local relapse, in four cases without metastatic disease. Two patients remain in complete remission 27 and 25 months after surgery. The median time to progression was shorter in patients presenting a cardiac angiosarcoma than other histological types (3 vs 14 months, $P < 0.01$). Twelve patients have died, with a median overall survival of 12 months. The 2-year survival rate is 26%. Survival was significantly longer for patients with completely resected tumours (22 vs 7 months; $P = 0.02$) and those who did not have angiosarcoma (18 vs 7 months; $P = 0.04$). In conclusion, post-operative conventional doxorubicin-based chemotherapy failed to modify the natural history of patients with resected cardiac sarcomas. Locoregional failure remains the main problem even after histologically complete resection. New approaches must be tested in patients with primary cardiac sarcoma.

**Keywords:** vascular sarcomas; cardiac sarcomas; cardiac tumours; chemotherapy; sarcomas

Primary cardiac sarcomas are extremely rare. They account for 8% of all primary cardiac tumours resected (Blondeau, 1990), but the necroscopic incidence is threefold that of operated cases (Chomette et al, 1985). All histological types have been described, and angiosarcoma, representing 30–45% of cases, is the most commonly described (McAllister et al, 1979; Silverman, 1980).

During the course of the disease, the presenting symptoms (dyspnoea, chest pain, congestive heart failure, palpitations, fever, or myalgia) appear late, and these delayed manifestations generally reflect a wide local extension with severe cardiac damage (Goodwin, 1968). Even though distant metastasis frequently occurs, the clinical course in patients with primary cardiac sarcoma is related to local tumour extension (Becker et al, 1985).

Since the first surgical resection of a cardiac tumour was reported (Crafoord, 1955), surgery has become the standard first-line treatment, able to provide substantial palliation of symptoms and prolong survival (Dein et al, 1987). However, most patients present with marginally resectable or technically non-resectable disease at diagnosis, and patients surviving 2 years after local excision are rarely reported (Murphy et al, 1990; Moggio et al, 1992).

Given the likely inadequacy of surgical margins and the high risk of distant metastasis, both adjuvant radiotherapy and systemic chemotherapy have been recommended. Unfortunately, the results of these multimodality therapies are disappointing. In two recent retrospective series (Putman et al, 1991; Burke et al, 1992), surgical resection was the only factor found to improve survival, with a suggested enhancement conferred by post-operative chemotherapy.

This study analyses the patterns of relapse and survival of 15 patients who have undergone resection of a primary cardiac sarcoma, together with an adjuvant anthracycline-based chemotherapy regimen at the same institution.

### MATERIAL AND METHODS

From October 1978 to December 1995, 19 patients were admitted to our institution with an initial diagnosis of primary malignant cardiac sarcoma. Patients were referred for additional treatment by French or Italian cardiovascular or thoracic institutions.

Histological diagnosis was made on the operative specimens and all slides were reviewed by the pathology department of the Institut Gustave Roussy, France. Diagnostic criteria excluded patients with a history of soft-tissue or bone sarcoma. Surgery with any macroscopic or microscopic involvement of margins was classified as incomplete. Complete resection was defined by the absence of involvement of all margins. The medical record of each patient was reviewed and routine demographic data recorded until 1 March 1998. Lost follow-up information on two patients was obtained by corresponding with their physicians.

Survival was calculated using the Kaplan–Meier method (Kaplan and Meier, 1958). Groups were compared using the generalized Wilcoxon–Gehan test (Gehan et al, 1965). All $P$-values resulted from two-sided tests. Time to progression was calculated from the date of surgical resection to the date of first relapse. Time

*Received 21 August 1997*
*Revised 7 May 1998*
*Accepted 19 May 1998*

*Correspondence to:* A Llombart-Cussac, Servicio Oncologia Medica, Instituto Valenciano de Oncologia, C/P Beltran Baguena 9, Valencia 46021, Spain

**Table 1** Patient population

| Patient no. | Age (years) | Sex | Histology | Complete resection at initial surgery | Post-operative chemotherapy | Disease-free survival (months) | Relapse | Survival (months) | Status |
|---|---|---|---|---|---|---|---|---|---|
| 1 | 39 | M | Lipo | No | AMV | 4 | L | 5 | D |
| 2 | 50 | F | AS | No | CYVADIC | 3 | L+M | 4 | D |
| 3 | 40 | M | AS | No | CYVADIC | 3 | M | 5 | D |
| 4 | 16 | M | STS | Yes | AI | 10 | L+M | 11 | D |
| 5 | 37 | M | AS | No | AI | 6 | M+L | 12 | D |
| 6 | 57 | M | AS | Yes | AI[c] | 12 | M+L | 22 | D |
| 7 | 66 | F | Leio | No | CYVADIC | 5 | L | 10 | D |
| 8 | 28 | M | Rabdo | Yes[a] | CYVADIC + A | 24 | L+M | 82 | A, WMD |
| 9 | 60 | M | AS | No | AI | 1 | L+M | 2 | D |
| 10 | 46 | M | MFH | No | A | 25 | L | 30 | D |
| 11 | 58 | M | MFH | No | AI | 14 | L+M | 18 | D |
| 12 | 38 | M | AS | No | AI | 4 | L+M | 7 | D |
| 13 | 51 | F | Leio | Yes[b] | AI | 11 | L | 13 | D |
| 14 | 43 | M | Rabdo | Yes | AI | 27 | – | 27 | A, NED |
| 15 | 56 | M | MFH | Yes | AI | 25 | – | 25 | A, NED |

[a]Patient had three resections; [b]patient had two resections; [c]radiation therapy also given. A, doxorubicin; AI, A + ifosfamide; CYVADIC, cyclophosphamide + DTIC + A + vincristine; AMV, A + methotrexate + V; AS, angiosarcoma; MFH, malignant fibrous histiocytoma; Lipo, liposarcoma; Leio, leiomyosarcoma; STS, synovial sarcoma. M, metastatic progression; L, local progression; D, dead; A, alive; WMD, with metastatic disease; NED, no evidence of disease.

to local failure was determined from the date of resection to the date of local relapse. Survival was calculated from the date of the first operation to the date of last follow-up information.

### RESULTS

Four patients with primary cardiac sarcoma (PCS) have been excluded from the analysis. Two of these had an unresectable tumour, suitable for biopsy only; death occurred 2 and 6 months after diagnosis. The other two patients presented with synchronous lung or cutaneous metastases. Local resection was complete in one, but incomplete in the other. Neither patient responded to an anthracycline-based chemotherapy regimen; in both cases the cause of death was local progression 5 and 12 months after surgery.

The characteristics of the remaining 11 male and four female patients are listed in Table 1. The median age was 45 years (range 16–66). Six patients (40%) had an angiosarcoma, three a malignant fibrous histiocytoma, two a leiomyosarcoma, two a rhabdomyosarcoma, and the remaining two had a liposarcoma and a synovialsarcoma. The chamber of origin was associated with the histological subtype: angiosarcoma commonly originated in the right side of the heart (right atrium, $n = 4$; left atrium, $n = 1$; right atrium and ventricle, $n = 1$). All the other histological types but one developed on the left side of the heart (left atrium, $n = 7$; left atrium and ventricle, $n = 1$; right atrium, $n = 1$). All patients presented symptoms at diagnosis; cardiac manifestations varied from chest pain to cardiac heart failure or constrictive pericarditis. One patient developed a tumour embolus as the first symptom. A patient with an implanted metallic mitral valve (patient 11) developed a malignant fibrous histiocytoma adjacent to this valve.

A complete resection was achieved in six patients, but was incomplete in nine (four with microscopical residual disease and five with gross residual tumour). Five out of six patients with an angiosarcoma and four out of nine with other histological types had incomplete resections.

After initial debulking surgery, all patients had a performance status (PS) of 0–1 (PS 0, eight patients; PS 1, seven patients) without severe congestive heart failure or obstructive symptoms, documented by a normal left ventricular ejection fraction (LVEF). Three out of four patients with gross residual tumour, along with seven other patients, underwent a chest scan or a cardiac magnetic resonance examination before chemotherapy. Adjuvant chemotherapy started within 6 weeks of surgery and included (Table 1): cyclophosphamide, doxorubicin, vincristine and dacarbazine (CYVADIC regimen, $n = 4$); ifosfamide and doxorubicin (AI regimen, $n = 9$); doxorubicin, methotrexate and vincristine (AMV regimen, $n = 1$); and doxorubicine alone ($n = 1$). The mean number of chemotherapy courses for the whole group was four (range 1–6). Chemotherapy was stopped in one patient after three cycles (cumulative doxorubicin dose 225 mg $m^{-2}$) because of impairment of LVEF. In five patients in whom resection was incomplete, a local and/or distant recurrence was observed during adjuvant chemotherapy. The nine patients who completed the planned chemotherapy received a median doxorubicin cumulative dose of 300 mg $m^{-2}$ (range 250–360 mg $m^{-2}$). Additional mediastinal adjuvant radiotherapy (50 Gy) was delivered to one patient in whom surgical resection was incomplete.

Twelve patients (80%) developed local recurrence, which was the first tumour event in eight of them, but in four was synchronous with metastatic progression. The median disease-free interval for local relapse was 11 months (range 1–25). Distant metastases were observed in nine patients after a median time of 7 months (range 1–65). The metastatic sites involved were the lung ($n = 7$), soft tissue ($n = 3$), bone ($n = 2$), liver ($n = 1$), abdomen ($n = 1$) and the central nervous system ($n = 1$). Only two patients were free of disease at the time of analysis, with a follow-up of 25 and 27 months. The median disease-free progression interval (DFI) was 10 months (range 1–25).

Patients with primary cardiac angiosarcoma had a significantly shorter DFI than patients with other histological types ($P < 0.05$); the median DFI was 4 months (range 1–12) and 13 months (range 4–25) for vascular and non-vascular sarcoma respectively.

At the first sign of relapse, different palliative procedures were implemented. Further chemotherapy regimens were administered

© Cancer Research Campaign 1998

**Figure 1** Overall survival

**Figure 2** Overall survival by histological type in five patients with measurable lesions (five second-line and two third-line regimens). One minor response (of less than 3 months) and six cases of progressive disease were observed. No improvement of symptoms was obtained with mediastinal radiotherapy in four patients who had local progression. Two patients underwent further surgery. One patient died during a heart transplant because of local relapse. Finally, one patient with a primary cardiac rhabdomyosarcoma obtained two remissions with local resections 24 and 43 months after the first surgical procedure; he developed a metastatic recurrence 65 months later, which was also completely resected. This young patient developed symptomatic congestive heart failure (LVEF = 37%) after three cardiac resections and 450 mg $m^{-2}$ of doxorubicin cumulative doses.

At the time of the analysis, 12 patients (80%) have died. The cause of death was related to locoregional progression in 11. One patient, who had no local recurrence, died of pulmonary metastases 5 months after surgery. The median overall survival was 12 months (range 2–68), and the 2-year overall survival rate was 19% (Figure 1).

Survival was significantly shorter in patients with angiosarcoma than in the rest of the population (7 vs 18 months, $P = 0.04$) as shown in Figure 2. The six patients with a completely resected cardiac sarcoma had a significantly longer survival rate than the nine with residual disease (Figure 3), with a median survival of 22 months and 7 months respectively ($P = 0.02$).

### DISCUSSION

Primary sarcoma of the heart is rare with a dismal short-term prognosis. Surgery is the only treatment capable of improving outcome even after palliative resection, but long-term survivors are still anecdotal. Sporadic cases of long-term survivors treated with radiation therapy (Stevens et al, 1992) and/or chemotherapy (Potter et al, 1989) have been reported. However, because of the small number of patients, we do not know whether post-surgical treatment can confer a gain in survival.

**Figure 3** Overall survival by quality of surgery

For common soft-tissue sarcomas, adjuvant anthracycline-containing chemotherapy regimens seem to decrease the incidence of local and metastatic relapse (Tierney et al, 1995). Inadequate resection margins, high-grade tumours and primary truncal sarcomas are unfavourable independent prognostic factors (Trojani et al, 1984) and they seem to be good indicators for adjuvant chemotherapy. Mainly for these reasons, most authors have recommended complementary chemotherapy with or without radiotherapy in patients with resected PCS (Demmy, 1996).

The goal of this retrospective study was to analyse the potential benefit of post-operative doxorubicin-containing chemotherapy in healthy patients with PCS having undergone local resection. Patients with unresectable tumours, low performance status or any contraindication to anthracyclines were excluded. Doxorubicin was used in most cases combined with ifosfamide or dacarbazine, two active regimens in advanced soft-tissue sarcomas (Antman et al, 1993; Edmonson et al, 1993; Santoro et al, 1995). Chemotherapy was started within 6 weeks of surgery. Thus, this series represents a selected group of PCS patients treated by surgery and optimal chemotherapy, with a hypothetically more favourable outcome than in any other published study. Despite this, results are disappointing. The median interval to first relapse (local or metastatic) was 10 months and the median survival was 12 months. Only three patients were alive at the time of analysis, one with metastatic disease. These results move towards those obtained by another series (Putman et al, 1989) who achieved an overall survival rate of 14% at 2 years in a less favourable population, including patients with non-resected or untreated disease.

The type of sarcoma was the only histological finding correlating with progression-free survival and overall survival. Patients with cardiac angiosarcoma had the least favourable outcome because all relapsed and died, 83% of them in the first year. None of the published series stressed this point, but Putman et al (1989) reported no surviving patients with angiosarcoma 18 months after diagnosis as opposed to 42% (5 out of 12 patients) with other histological types. In common sarcomas (visceral or soft tissue), angiosarcomas have a poor prognosis irrespective of primary site and histological grade. A retrospective analysis on 1100 patients with resected sarcoma (Hashimoto et al, 1992) reported an overall survival of 14% at 5 years for angiosarcoma, as opposed to 70% for patients with myxoid liposarcoma. In PCS, a previous study (Burke et al, 1991) demonstrated a relationship between histological features, for example high mitotic activity or tumour necrosis and dismal outcome.

© Cancer Research Campaign 1998

The quality of initial tumour debulking was predictive of a disease-free and status survival in our study. One series (Putnam et al. 1991) reported an overall survival of 24 months for patients having undergone wide resection of tumours, compared with only 10 months in all other patients. Similarly, in the univariate analysis, complete or wide excision was associated with an increased survival in the Armed Forces Institute series (Burke et al. 1992)

The extent of the surgical procedure seemed related to histological type because angiosarcoma patients had more incomplete resections (five out of six), whereas patients with non-vascular sarcomas had more completely resected tumour (five out of nine). This point did not reach statistical significance probably because of the small number of patients ($P = 0.1$). However, the relatively high incidence of pericardic involvement and multifocal presentation of angiosarcomas could partially account for the inadequate local control of these tumours.

Local relapse was the first and most common type of recurrence. Only one patient, who died early because of pulmonary metastases, had no local failure. However, distant metastases were generally detected at the same time or a few months after local recurrence. The same pattern of relapse is observed in patients with marginally or incompletely resected high-grade soft-tissue sarcomas (Choong et al. 1995; Lewis et al. 1997).

Primary cardiac sarcomas can be divided into two groups according to the histological subtype. Cardiac angiosarcomas tend to arise in the right chambers of the heart, thus delaying symptoms (Janigan et al. 1986). They are initially larger tumours and carry a poor prognosis whatever the quality of initial surgical resection, which is generally incomplete (Potter et al. 1989). These factors, compounded by the high frequency of distant metastasis at diagnosis (Herrmann et al. 1992), contribute to the limited duration of survival. Moreover, these tumours are usually resistant to conventional cytotoxic agents as observed in our patients. New effective drugs, and innovative strategies such as inmunotherapy, antiangiogenic factors or intensive chemotherapy regimens need to be tested on these high-grade aggressive tumours.

In contrast, non-vascular cardiac sarcomas appear to be less aggressive; they arise predominantly in the left chambers of the heart and present as an intracavity mass. The most frequent cause of relapse in our series was local progression. These tumours should undergo optimal resection, and neoadjuvant chemotherapy could be incorporated in treatment. Adjuvant radiotherapy seems mandatory if the aim is to preclude local relapse, but the best option for selected patients could be an orthotopic cardiac transplant.

In recent years, orthotopic heart transplant has been proposed to only a few unselected patients with primary cardiac sarcoma (Goldstein et al. 1995), namely patients with locally advanced tumours (Siebenmann et al. 1990) or angiosarcoma (Armitage et al. 1990; Crespo et al. 1993). Most of these cases rapidly developed distant metastases thereafter, and long-term survivors are still rare (Aravot et al. 1989). Based on our data, heart transplantation can be proposed to patients with widely resected non-angiosarcoma cardiac tumours with no obvious distant metastasis.

In conclusion and despite the dismal prognosis, a benefit in quality of life and survival has been observed over the last decade in patients with PCS. This improvement is based on (i) the routine use of echocardiography, allowing a more accurate diagnosis; (ii) the development of new surgical techniques which have improved the quality and number of cardiac resections (Perchinsky et al. 1997); and, perhaps, (iii) the optimization of chemotherapy regimens including anthracyclins and high-dose ifosfamide (Le Cesne et al. 1996).

In the next years, oncologists will be concerned about additional treatments in patients after optimal PCS resection. The impact of conventional chemotherapy cannot be determined in this small and retrospective study, but a need for more active systemic treatments and/or new therapeutic approaches emerged from the outcome of these patients.

### REFERENCES

Antman K, Crowley J, Balcerzak SP, Rivkin SE, Weiss GR, Elias A, Natale RB, Cooper RM, Barlogie B, Trump DL, Doroshow JH, Aisner J, Pugh RP, Weiss RB, Cooper BA, Clamond GH and Baker LH (1993) An intergroup phase III randomized study of doxorubicin and dacarbacine with or without ifosfamide and mesna in advanced soft tissue and bone sarcomas. *J Clin Oncol* **11**: 1276–1285

Aravot DJ, Banner NR, Madden B, Aranki S, Khaghani A, Fitzgerald M, Radley-Smith R and Yacoud MH (1989) Primary cardiac tumors – is there a place for cardiac transplantation? *Eur J Cardiothoracic Surg* **3**: 521–524

Armitage JM, Kormos RL, Griffith BP, Fricker FJ and Hardesty RL (1990) Heart transplantation in patients with malignant disease. *J Heart Transplant* **9**: 627–629

Becker RC, Loeffler JS, Leopold KA and Underwood DA (1985) Primary tumors of the heart: a review with emphasis on diagnosis and potential treatment modalities. *Semin Surg Oncol* **1**: 161–170

Blondeau PH (1990) Primary cardiac tumors: French studies of 533 cases. *Thorac Cardiovasc Surg* **38**: 192–195

Burke AP, Cowan D and Virmani R (1992) Primary sarcomas of the heart. *Cancer* **69**: 387–395

Chomette G, Auriol M, Cabrol C and Tranbaloc P (1985) Primary malignant tumors of the heart: anatomo-clinical study of 12 cases. *Ann Med Interne* **136**: 301–305

Choong PF, Gustafson P and Rydholm A (1995) Size and timing of local recurrence predicts metastasis in soft tissue sarcoma. Growth rate index retrospectively analyzed in 134 patients. *Acta Orthop Scand* **66**: 147–152

Crafoord C (1955) Cancer report in international symposium. Fascicle *Cardiovascular Surgery*. Henry Ford Hospital: Detroit

Crespo MG, Pulpon LA, Gonzalo-Pradas F, Serrano S, Segovia J, Vegazo I, Salas C, Espana P, Silva L, Burgos R, Tellez G and Figuera D (1993) Heart transplantation for cardiac angiosarcoma: should its indication be questioned? *J Heart Lung Transplant* **12**: 527–530

Dein JR, Frist WH, Stinson EB, Stinson EB, Miller D, Baldwin J, Oyer P, Jamieson S, Mitchell RS and Shumway N (1987) Primary cardiac neoplasms, early and late results of surgical treatment in 42 patients. *J Thorac Cardiovasc Surg* **93**: 502–511

Demmy TD (1996) Tumors of the heart and pericardium. In *Comprehensive Textbook of Thoracic Oncology*. Aisner J, Arriagada R, Green M, Martini N and Perry MC (eds), pp. 695–710. Williams and Wilkins Warerly: Baltimore

Edmonson JH, Ryan LM, Blum RH, Brooks JSJ, Shiraki M, Frytak S and Parkinson DR (1993) Randomized comparison of doxorubicin alone versus ifosfamide plus doxorubicin or mytomicin, doxorubicin, and cisplatin against advanced soft tissue sarcomas. *J Clin Oncol* **11**: 1269–1275

Gehan EA (1965) A generalized wilcoxon test for compating arbitrarily singly-censored samples. *Biometrika* **52**: 203–223

Goldstein DJ, Oz MC, Rose EA, Fisher P and Michler RE (1995) Experience with heart transplantation for cardiac tumors. *J Heart Lung Transplant* **14**: 382–386

Goodwin JF (1968) The spectrum of cardiac tumors. *Am J Cardiol* **21**: 307–314

Hashimoto H, Daimarou Y, Takeshita S, Tsuneyoshi M and Enjoji M (1992) Prognostic significance of histologic parameters of soft tissue sarcomas. *Cancer* **70**: 2816–2821

Herrmann MA, Shankerman RA, Edwards WD, Shub C and Schaff HV (1992) Primary cardiac angiosarcoma: a clinicopathologic study of six cases. *J Thorac Cardiovasc Surg* **103**: 655–664

Janigan DT, Husain A and Robinson NA (1986) Cardiac angiosarcomas: a review and a case report. *Cancer* **57**: 852–859

Kaplan EL and Meier P (1958) Nonparametric estimation from incomplete observations. *J Am Stat Assoc* **53**: 457–481

Le Cesne A, Antoine E, Spielmann M, Le Chevalier T, Brain E, Toussaint C et al (1995) High-dose ifosfamide: circumvention of resistance to standard-dose ifosfamide in advanced soft tissue sarcomas. *J Clin Oncol* **13**: 1600–1608

Lewis JJ, Leung D, Heslin M, Woodruff JM and Brennan MF (1997) Association of local recurrence with subsequent survival in extremity soft tissue sarcoma. *J Clin Oncol* **15**: 646–652

McAllister Jr HA and Fenoglio Jr JJ (1978) Tumors of the cardiovascular system. In *Atlas of Tumor Pathology*, fascicle 15, pp. 81–88. Armed Forces Institute of Pathology, Washington DC

Moggio RA, Pucillo AL, Schechter AG, Pooley RW, Sarabu MR and Reed GE (1992) Primary cardiac tumors. Diagnosis and management in 14 cases. *NY State J Med* **92**: 49–52

Murphy MC, Sweeney MS, Putnam Jr JB, Walker WE, Frazier OH, Ott DA and Cooley DA (1990) Surgical treatment of cardiac tumors: a 25-year experience. *Ann Thorac Surg* **49**: 612–617

Perchinsky MJ, Lichtenstein SV and Tyers GF (1997) Primary cardiac tumors. Forty years' experience with 71 patients. *Cancer* **79**: 1809–1815

Potter R, Baumgart P, Greve H and Schnepper E (1989) Primary angiosarcoma of the heart. *Thorac Cardiovasc Surg* **37**: 374–378

Putnam Jr JB, Sweeney MS, Colon R, Lanza LA, Frazier OH and Cooley DA (1991) Primary cardiac sarcomas. *Ann Thorac Surg* **51**: 906–910

Santoro A, Tursz T, Mouridsen H, Verweij J, Steward W, Somers R, Buesa J, Casali P, Spooner D, Rankin E, Kirkpatrik A, Van Glabbeke M and Van Oosterom (1995) Doxorubicin versus CYVADIC versus doxorubicin plus ifosfamide in first-line treatment of advanced soft tissue sarcomas: a randomized study of the European organization for research and treatment of cancer soft tissue and bone sarcoma group. *J Clin Oncol* **13**: 1537–1545

Siebenmann R, Jenni R, Makek M, Oelz O and Turina M (1990) Primary synovial sarcoma of the heart treated by heart transplantation. *J Thorac Cardiovasc Surg* **99**: 566–567

Silverman NA (1980) Primary cardiac tumors. *Ann Surg* **191**: 127–138

Stevens CW, Sears-Rogan P, Bitterman P and Torrisi J (1992) Treatment of malignant fibrous histiocytoma of the heart. *Cancer* **69**: 956–961

Tierny JF, Mosseri V, Stewart LA, Souhami RL and Palmer MKB (1995) Adjuvant chemotherapy for soft tissue sarcoma: review and meta-analysis of the published results of randomised clinical trials. *Br J Cancer* **72**: 469–475

Trojani M, Contesso G, Coindre JM, Rouesse J, Bui NB, De Mascarel A, Goussot JF, David M, Bonichon F and Lagarde C (1984) Soft-tissue sarcomas of adults: study of pathological prognostic variables and definition of a histopathological grading system. *Int J Cancer* **33**: 37–42

*© Cancer Research Campaign 1998*